# Supplementary material for: Reconciling Mining with the Conservation of Cave Biodiversity: A Quantitative Baseline to Help Establish Conservation Priorities
Source: PLoS One. 2016 Dec 20;11(12):e0168348. doi: 10.1371/journal.pone.0168348 (PMC5173368; doi:10.1371/journal.pone.0168348)
Supplement: S1 Dataset — (ZIP) [file pone.0168348.s002.zip › Taxa/Serra Norte/SN_2013/N4E/Lista N4E-02.pdf]

CAVIDADE N4E-0002

Classe	Ordem	Fam/Outros	Gên/Outros	Espécie	S-U	U-S	Zon.
Arachnida	Acari	Argasidae	*Ornithodoros*	sp.1		X	P
Arachnida	Acari	Ixodidae	*Amblyomma*	*ovale*	X		
Arachnida	Acari	Prostigmata		jovem	X		
Arachnida	Amblypygi	Charinidae	*Charinus*	sp.1		1	P
Arachnida	Araneae	Araneidae	*Alpaida*	sp.1	X		
Arachnida	Araneae	Mysmenidae	*Microdipoena*	sp.1		X	E
Arachnida	Araneae	Ochyroceratidae		jovem		X	E/P
Arachnida	Araneae	Pholcidae	*Mesabolivar*	sp.1	X	X	E
Arachnida	Araneae	Salticidae		sp.	X	X	E
Arachnida	Araneae	Scytodidae	*Scytodes*	sp.	X	1	P
Arachnida	Araneae	Tetragnathidae	*Azilia*	sp.	X		
Arachnida	Araneae	Tetragnathidae	*Leucauge*	sp.2		X	P
Arachnida	Araneae	Theridiidae		jovem		X	E
Arachnida	Araneae	Theridiosomatidae	*Plato*	sp.1	X	X	E/P
Arachnida	Araneae	Uloboridae	*Miagrammopes*	sp.	X		
Arachnida	Opiliones	Escadabiidae		sp.2		X	P
Arachnida	Opiliones	Sclerosomatidae	*Prionostema*	sp.1		2	E
Arachnida	Pseudoscorpiones	Chernetidae	*Spelaeochernes*	sp.1	X	X	E/P
Chilopoda	Scolopendromorpha	Cryptopidae	*Cryptops*	jovem		1	P
Chilopoda	Scutigeromorpha	Pselliodidae	*Sphendononema*	*guildingii*	X		
Diplopoda	Polydesmida	Chelodesmidae		sp.	X		
Diplopoda	Polydesmida	Pyrgodesmidae		sp.1	X	X	P
Entognatha	Collembola	Isotomidae		sp.1		X	P
Entognatha	Collembola	Paronellidae		sp.1		X	E
Entognatha	Collembola	Paronellidae		sp.4		X	P
Entognatha	Collembola			sp.	X		
Entognatha	Diplura	Campodeidae		sp.1	X		
Insecta	Blattodea			jovem	X	1	E
Insecta	Coleoptera	Curculionidae	Scolytinae	sp.	X		
Insecta	Coleoptera	Staphylinidae	Pselaphinae	sp.	X		
Insecta	Diptera	Cecidomyiidae	Cecidomyiinae	sp.		X	E
Insecta	Diptera	Chironomidae		sp.	X	X	E
Insecta	Diptera	Culicidae	*Culex*	sp.		X	E
Insecta	Diptera	Psychodidae	Phlebotominae	sp.		X	E/P
Insecta	Diptera	Psychodidae	*Sciopemyia*	*sordellii*	X		
Insecta	Heteroptera	Cydnidae		sp.	X	X	E
Insecta	Heteroptera	Nabidae		sp.		X	P
Insecta	Heteroptera	Reduviidae	Emesinae	sp.8		1	E
Insecta	Heteroptera	Reduviidae	Reduviinae	sp.		1	P
Insecta	Hymenoptera	Diapriidae		sp.1		X	P
Insecta	Hymenoptera	Formicidae	*Brachymyrmex*	sp.	X		
Insecta	Hymenoptera	Formicidae	*Camponotus*	sp.	X		
Insecta	Hymenoptera	Formicidae	*Dolichoderus*	aff. *bispinosus*	X		
Insecta	Hymenoptera	Formicidae	*Eurhopalothrix*	sp.1		X	P
Insecta	Hymenoptera	Formicidae	*Pachycondyla*	*constricta*	X		
Insecta	Hymenoptera	Formicidae	*Pachycondyla*	sp.2		X	E
Insecta	Hymenoptera	Formicidae	*Paratrechina*	sp.	X		
Insecta	Hymenoptera	Formicidae	*Trachymyrmex*	sp.1		X	P
Insecta	Isoptera	Termitidae	*Nasutitermes*	sp.		X	P
Insecta	Lepidoptera	Tineoidea		jovem	X		
Insecta	Orthoptera	Phalangopsidae	*Phalangopsis*	sp.	X		
Insecta	Psocoptera			jovem	X	X	E/P
Insecta	Thysanura	Ateluridae		jovem		X	P
Insecta	Thysanura	Nicoletiidae		sp.1		X	P
Gastropoda	Pulmonata	Subulinidae	*Leptinaria*	sp.	X		

Gastropoda	Pulmonata	Systrophiidae	*Happia*	sp.	X		
Malacostraca	Isopoda	Philosciidae		sp.	X		
Mammalia	Chiroptera	Emballonuridae	*Peropteryx*	*kappleri*		2	E/P
Mammalia	Chiroptera	Phyllostomidae	*Carollia*	*perspicillata*		60	E/P
Mammalia	Chiroptera	Phyllostomidae	*Glossophaga*	*soricina*		2	E/P
